# Integrated genetic analyses revealed novel human longevity loci and reduced risks of multiple diseases in a cohort study of 15,651 Chinese individuals

**DOI:** 10.1111/acel.13323

**Published:** 2021-03-03

**Authors:** Xiaomin Liu, Zijun Song, Yan Li, Yao Yao, Mingyan Fang, Chen Bai, Peng An, Huashuai Chen, Zhihua Chen, Biyao Tang, Juan Shen, Xiaotong Gao, Mingrong Zhang, Pengyu Chen, Tao Zhang, Huijue Jia, Xiao Liu, Yong Hou, Huanming Yang, Jian Wang, Fudi Wang, Xun Xu, Junxia Min, Chao Nie, Yi Zeng

**Affiliations:** ^1^ BGI‐Shenzhen Shenzhen China; ^2^ China National Genebank Shenzhen China; ^3^ BGI Education Center University of Chinese Academy of Sciences Shenzhen China; ^4^ The First Affiliated Hospital Institute of Translational Medicine School of Medicine, Zhejiang University Hangzhou China; ^5^ Center for the Study of Aging and Human Development Medical School of Duke University Durham USA; ^6^ Center for Healthy Aging and Development Studies National School of Development, Raissun Institute for Advanced Studies, Peking University Beijing China; ^7^ School of Labor and Human Resources Renmin University Beijing China; ^8^ Beijing Advanced Innovation Center for Food Nutrition and Human Health China Agricultural University Beijing China; ^9^ Business School of Xiangtan University Xiangtan China; ^10^ BGI Genomics BGI‐Shenzhen Shenzhen China; ^11^ Guangdong Provincial Key Laboratory of Genome Read and Write Shenzhen China

**Keywords:** centenarians, human genetics, life span, longevity, sex difference

## Abstract

There is growing interest in studying the genetic contributions to longevity, but limited relevant genes have been identified. In this study, we performed a genetic association study of longevity in a total of 15,651 Chinese individuals. Novel longevity loci, *BMPER* (rs17169634; *p* = 7.91 × 10^−15^) and *TMEM43/XPC* (rs1043943; *p* = 3.59 × 10^−8^), were identified in a case–control analysis of 11,045 individuals. *BRAF* (rs1267601; *p* = 8.33 × 10^−15^) and *BMPER* (rs17169634; *p* = 1.45 × 10^−10^) were significantly associated with life expectancy in 12,664 individuals who had survival status records. Additional sex‐stratified analyses identified sex‐specific longevity genes. Notably, sex‐differential associations were identified in two linkage disequilibrium blocks in the *TOMM40/APOE* region, indicating potential differences during meiosis between males and females. Moreover, polygenic risk scores and Mendelian randomization analyses revealed that longevity was genetically causally correlated with reduced risks of multiple diseases, such as type 2 diabetes, cardiovascular diseases, and arthritis. Finally, we incorporated genetic markers, disease status, and lifestyles to classify longevity or not‐longevity groups and predict life span. Our predictive models showed good performance (AUC = 0.86 for longevity classification and explained 19.8% variance of life span) and presented a greater predictive efficiency in females than in males. Taken together, our findings not only shed light on the genetic contributions to longevity but also elucidate correlations between diseases and longevity.

## Graphical abstract

This genetic association study of longevity on 15,651 Chinese individuals (3,448 nonagenarians and 2,509 centenarians) identified two novel human longevity loci. Two novel longevity loci, *BMPER* (rs17169634; *p* = 7.91 × 10^−15^) and *TMEM43/XPC* (rs1043943; *p* = 3.59 × 10^−8^) reached genome‐wide significance. Interestingly, gender differential associations revealed two linkage disequilibrium blocks on *TOMM40/APOE* region. In addition, our predictive models showed good performance (AUC = 0.86 for longevity classification and explained 19.8% variances of life span), and higher efficiencies in females than males. These findings provide potential strategies for improving healthy aging.

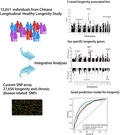

## 1 INTRODUCTION

The average human life expectancy has been rising for decades (Greene, 2001; Oeppen & Vaupel, 2002), and it was recently estimated that the number of long‐lived individuals (more than 90 years old) was 63.5 million worldwide as of 2020 (United Nations, 2019). It is clear that longevity represents a complex trait that is influenced by genetic and environmental factors and their interactions (Passarino et al., 2016). Twin studies (Herskind et al., 1996; Skytthe et al., 2003) have estimated that the heritability of longevity is approximately 20%–30% in modern societies, and the proportion increases to approximately 40% for long‐lived individuals (Hjelmborg et al., 2006; Perls et al., 2000; Terry et al., 2007; van den Berg et al., 2019). Although longevity is considered to exhibit relatively high heritability, limited genetic loci related to this trait have been identified in previous genome‐wide association studies (GWAS; Deelen et al., 2014; Joshi et al., 2017; McDaid et al., 2017; Sebastiani et al., 2012; Zeng et al., 2016). Apolipoprotein E (*APOE*) is the only gene that has been replicated by multiple independent GWAS meta‐analyses (Deelen et al., 2019; Joshi et al., 2017; McDaid et al., 2017). One recent meta‐analysis revealed rs7676745 near *GPR78* as a novel locus (Deelen et al., 2019). In European populations, other GWAS meta‐analysis studies have replicated several longevity genes, including *CHRNA3/5*, *CDKN2A/B*, *SH2B3*, and *FOXO3A* (Joshi et al., 2017; McDaid et al., 2017). Our previous GWAS in a Chinese population additionally identified *IL6* and *ANKRD20A9P* (Zeng et al., 2016). One possible reason for the lack of replication could be the variation in phenotype definitions. Some studies have compared old cases with young controls. The selection of age cutoffs varies among different studies. A recent study conducted on multi‐ethnic datasets used the 90th/99th survival percentile as the age cutoff (Deelen et al., 2019). Some other studies have used more extrema cutoffs, with only centenarians being included among the cases (Sebastiani et al., 2012, 2017; Zeng et al., 2016). The use of different definitions for cases and controls may lead to heterogeneity. Continuous phenotypes have also been widely used for longevity genetic studies. A person's life span is the most obvious phenotype. Nevertheless, many ongoing cohorts contain younger participants, resulting in the limited sample size of people with exact death dates. Therefore, the parental life span has been used as an alternative phenotype (McDaid et al., 2017; Timmers et al., 2019). The predicted life span based on family history life span has also been used (Yashin et al., 2018). However, because the lifestyles, health care, and other environmental factors are quite different between generations, the use of parental life spans might introduce substantial bias. Notably, the previous studies often focused on Western populations; very few studies have focused on Asians, which account for 42% of the long‐lived population worldwide (United Nations, 2019). The Chinese Longitudinal Healthy Longevity Survey (CLHLS) encompasses a nationwide sample of long‐lived adults and the “young old” in China, with up to 20 years of follow‐up which enable us to track longevity and mortality. Therefore, CLHLS can provide an ideal dataset in analyzing the association of genetic and non‐genetic data with life span in humans.

In addition to studying the genetics of longevity and life span, age‐related diseases and their correlations with longevity have attracted much attention (Sakaue et al., 2020). In either human centenarians or long‐lived animals, it has long been observed that longevity and the occurrence of diseases, such as cardiovascular and cerebral stroke, are inversely correlated either genetically or experimentally (Altmann‐Schneider et al., 2013; Hammond et al., 1971; Rosa et al., 2019; van der Lee et al., 2019). A previous study, using genetic data of parental life span, reported genetic correlations between several complex traits and mortality in a general population of European ancestry (Joshi et al., 2017). Our study defined individuals with ages greater than 90 as the longevity group, rather than the parental survival which is a debatable longevity phenotype. Therefore, a systematic exploration of the correlation between longevity and complex diseases in the current study may reveal more information.

Another research interest is to predict longevity and life span based on age‐related diseases and genetic markers. The polygenic risk score (PRS) generated from the summary statistics of association studies is a commonly used predictor for genetic factors. For example, a recent genetic study reported that a polygenic score could identify people with the top 10% parental survival PRS, who might outlive an average of 5 years those with the bottom 10% parental survival PRS (Timmers et al., 2019). In addition, circulating glucuronic acid levels (Ho et al., 2019) or telomeres (Whittemore et al., 2019) have been used as biomarkers for life span prediction. To date, there are very few studies that explore the potential of life span prediction by using a combination of genetic data, disease conditions, and lifestyle factors.

Here, we performed a large‐scale integrated analysis based on 15,651 Chinese individuals from CLHLS to identify the longevity genes, to explore the relationships between diseases and longevity, and to apply these longevity‐related factors for life span prediction. This study including 2,509 centenarians is one of the largest centenarian studies in the world (Deelen et al., 2019; Sebastiani et al., 2017; Timmers et al., 2019). We firstly designed a customized SNP chip using a carefully selected set of SNPs that captured 27,656 candidate variants correlated with longevity, age‐related diseases, and immunity. Next, we carried out a candidate‐gene association analysis on the age‐stratified phenotype (“cases” were defined as individuals surviving 90 years or over, while the “controls” had an age of less than 75, which is the average life span in China) and life span, respectively. Then, we performed a meta‐analysis incorporating the current dataset and our previously published GWAS dataset by removing the overlapped samples. Moreover, we evaluated the polygenic prediction of diseases on longevity using polygenic risk score (PRS) analysis and inferred causal relationships between longevity and diseases using the bidirectional Mendelian randomization method. Finally, we built predictive models for longevity, and life span by integrating genetic factors, disease status, and lifestyles. Overall, this study aimed to reveal the sex‐combined and sex‐specific longevity genes/pathways and investigate their predictive effectiveness on longevity and life span.

## 2 MATERIALS AND METHODS

### 2.1 Participants and phenotypes

This study included a total of 15,651 individuals from the Chinese Longitudinal Healthy Longevity Surveys (CLHLS), which were conducted in 1998, 2000, 2002, 2005, 2008, 2011, and 2014 in a randomly selected half of the counties and cities in 22 out of 31 provinces in China. The primary dataset (dataset 1) included 13,228 individuals with ages ranging from 30 to 114. All individuals were genotyped by using a well‐designed customized chip targeting approximately 27 K longevity‐related SNPs. These candidate SNPs were selected based on previously published associations with longevity, chronic diseases, and health indicators. For replication purposes, a dataset 2 included 4477 individuals based on our previous study (Zeng et al., 2016). 2054 samples were overlapped between the two datasets.

Demographic and clinical information (i.e., diseases) was recorded for participants in this study. Phenotypic data were collected using internationally standardized questionnaires adapted to the Chinese cultural and social context. The CLHLS study was approved by the Biomedical Ethics Committee of Peking University (IRB00001052‐13074). All participants or their legal representatives signed written consent forms in the baseline and follow‐up surveys.

### 2.2 Customized SNP chip design

We customize a SNP chip containing 27,656 selected longevity and disease‐related SNPs for targeted genotyping (Table S1). The selected SNPs could be characterized as corresponding to five major components (Tables S2 and S3): (1) 11,893 SNPs associated with longevity based on our previous CLHLS GWAS study on 4477 Chinese individuals (Zeng et al., 2016); (2) 1881 reported longevity SNPs based on the other previously published longevity studies, including the European Union (EU) longevity (Deelen et al., 2014) & New England centenarian study (NECS; Sebastiani et al., 2012), the Long Life Family Study (LLFS; Bae et al., 2013), and the Framingham Heart Study (FHS; Lunetta et al., 2007); (3) 3966 reported SNPs associated with diseases in the NHGRI‐EBI GWAS catalog (MacArthur et al., 2017); (4) 7260 SNPs associated with health indicators in the CLHLS and the Health and Retirement Study (HRS; Tanaka et al., 2017); and (5) 2656 tagging SNPs for imputing the alleles in the human major histocompatibility complex (MHC) region.

### 2.3 Sample filtering

The samples were required to meet 3 selection criteria: (1) a genotype calling rate >90%; (2) no existing population stratification according to a multidimensional scaling (MDS) procedure implemented in PLINK v1.07, based on which individuals deviating from the main population cluster were removed; and (3) no inclusion of duplicates or first‐degree relatives when evaluating pairwise through identity by descent (IBD). After sample filtering, 12,664 samples were included in the dataset 1.

### 2.4 Variant filtering

To determine the high‐quality genotypes, we applied a conservative inclusion threshold for variants: (1) minor allele frequency >5%, (2) genotype calling rate >90%; and (3) Hardy–Weinberg equilibrium (HWE) *p* > 10^−5^. To further confirm the quality of the genotypes, we calculated the concordance rate of the genotypes using 2,054 samples that overlapped between dataset 1 and dataset 2. Then, we removed the variants with a concordance rate <0.9 (Figure S7), which largely eliminated the bias caused by two different arrays (Illumina ZhongHua and Affix arrays). After variant filtering, 23,769 out of the 27,656 variants remained in dataset 1, and 818 K out of the 900 K variants remained in dataset 2.

### 2.5 Imputation

We performed imputation analysis by pre‐phasing genotypes with SHAPEIT v2.5 (Delaneau et al., 2011), and then imputing variants from the 1000 Genomes Project released on October 2014 with 2504 samples (http://1000genomes.org) as a reference panel using IMPUTE2 v2.3.1(Howie et al., 2009). SNPs with a quality score (*R* ^2^) >0.9 were included after imputation. After further quality control filtering for SNPs as described above, we eventually obtained 287 K SNPs from 12,664 individuals in dataset 1 and 5.6 M SNPs from 4,477 individuals in dataset 2 for the subsequent genetic association analyses.

### 2.6 MHC analysis

To identify potential MHC associations for longevity, 2,656 MHC tag SNPs were included in the 27 K arrays for dataset 1. Then, we used beagle 5 (Browning et al., 2018) with the HAN‐MHC datasets as a reference panel to impute MHC alleles, and the imputation accuracy was 0.96 at the two‐digit level as previously described (Zhou et al., 2016). In dataset 2, the samples were genotyped using Illumina HumanOmniZhongHua‐8 BeadChips tagging 900,015 SNPs, among which 8,350 SNPs were located in the MHC region. We imputed the MHC alleles using the same procedure applied for dataset 1 and obtained 104 imputed HLA alleles presented in both two datasets. For each dataset, 104 tests were performed in the cases and controls. In each test, one allele was compared with the other 103 alleles grouped together. The allelic 2 × 2 contingency table for a specific HLA allele contained the counts of that allele and the counts of the other 103 alleles in cases and controls. We next performed a meta‐analysis of the two datasets for the 104 imputed HLA alleles for longevity. Finally, a Bonferroni‐corrected *p* < 0.0005 = 0.05/104 for 104 alleles was defined as significant.

### 2.7 Association analysis for longevity

We performed genetic association analysis of 287 K imputed SNPs in dataset 1. More specifically, after sample filtering, a total of 8,490 individuals (4,662 cases with an age ≥90 and 3,828 controls with an age <75; 75 is the average life span of Chinese individuals) in dataset 1 were used for a case–control association analysis. We then performed association analysis in dataset 2 (Zeng et al., 2016). Since 1,922 individuals in dataset 2 were overlapped with 8,490 case/control samples in dataset 1, a case–control association analysis was performed in 2,555 independent samples by removing the 1,922 overlapped samples from 4,477 samples of dataset 2. The 2,555 independent samples included 1,105 centenarians’ cases and 1,450 controls with age <65. For each dataset, we applied logistic regression to calculate the *p*‐values and odds ratio (ORs) of the SNPs by adjusting for sex and the top two MDS dimensions using PLINK 1.07. Next, a meta‐analysis was performed on the two case–control association results, using inverse‐variance weighted fixed‐effect meta‐analysis in METAL software (https://genome.sph.umich.edu/wiki/METAL). To further replicate the results, we also reviewed association results from previous literatures, including EU & NECS, LLFS & FHS longevity GWAS, and studies in the GWAS catalog.

To investigate the correlations between the identified longevity‐related SNPs and diseases and the other traits, we reviewed diseases GWAS in this study (see Section 2.12 below). Then, we downloaded the summary statistics data from the Japan BioBank, a study of 300,000 Japanese citizens suffering from cancers, diabetes, rheumatoid arthritis, and other common diseases (Triendl, 2003). Similarly, we searched the longevity SNPs in the summary statistics data from Japan Biobank to examine their associations with metabolic traits and diseases.

### 2.8 Sex‐specific association analysis for longevity

We performed sex‐specific genetic association analyses in males and females separately. Male‐specific variants were identified as those that (1) were significantly associated with longevity in males (*p*_male_ < 5 × 10^−8^) but not significant in females (*p*_female_ > 0.05), and (2) exhibited a nominally significant sex difference (*p*‐value testing for difference in sex‐specific effect estimates, *p*_difference_ < 0.05; formula 1). Female‐specific variants were identified as (1) significantly associated with longevity in females (*p*_female_ < 5 × 10^−8^) but not significant in males (*p*_male_ > 0.05), and (2) exhibited a nominally significant sex difference (*p*_difference_ < 0.05; formula 1). For each variant, we calculated *p*_difference_ testing for the difference between the male‐specific and female‐specific beta estimates $\beta_{m}$ and $\beta_{f}$ using the T‐statistic (formula (1))

(1) $$T=\frac{\left(\beta_{m}‐\beta_{f}\right)}{\sqrt{{\text{SE}}_{m}^{2}+{\text{SE}}_{f}^{2}‐2*\text{cor}\left({\text{β}}_{m},\;{\text{β}}_{f}\right)*{\text{SE}}_{m}*{\text{SE}}_{f}}}$$

where ${\text{SE}}_{m}$ and ${\text{SE}}_{f}$ are the standard errors of the beta estimations in different sex groups.

### 2.9 Functional annotation and enrichment analysis

The significant loci with *p* < 10^−5^ identified in the sex‐combined genetic association analysis with longevity were mapped to genes using SNP2GENE in FUMA (Watanabe et al., 2017; http://fuma.ctglab.nl/). We mapped variants to genes based on physical distance within a 20‐kb window by the positional mapping method. The mapped genes were further investigated using the GENE2FUNC procedure, which provides hypergeometric tests for the list of enriched mapping genes in 53 GTEx tissue‐specific gene expression sets, 7,246 MSigDB gene sets, and 2,195 GWAS catalog gene sets. Using the GENE2FUNC procedure, we examined whether the mapped genes were enriched in specific diseases or traits in the GWAS catalog as well as whether enriched in specific Gene Ontology (GO) and Kyoto Encyclopedia of Genes (KEGG) categories. Significant results were selected if a false discovery rate (FDR)‐corrected *p* < 0.05 was observed.

To identify sex‐specific longevity pathways, the best‐fit *p*‐value cutoffs of 5 × 10^−5^ and 0.0015 (calculated by using PRSice; Euesden et al., 2015, software with the BEST FIT command) were used to select SNPs in females and males, respectively. SNPs were annotated and enriched for the pathways obtained from MSigDB version 5.2 limited to the KEGG, Reactome, and GO databases. A SNP was annotated to a gene if it fell within the interval of the coding sequence with the upstream and downstream 50 kb flanking regions. We performed the gene set enrichment analysis using MAGMA (de Leeuw et al., 2015) and sex‐specific pathway gene sets, and those showing a FDR‐corrected *p* < 0.05 were regarded as significant.

### 2.10 Observational correlation analysis

We had detailed questionnaire information including sex, age, diseases, cognition, and lifestyle factors. The observational correlation analysis was performed to assess the statistical relationship (i.e., the correlation) between longevity and these influencing factors, which were evaluated by multivariable linear regression analysis while adjusted for sex and the top two MDS (Table S13). In the multivariable linear regression model, the phenotype is longevity trait with longevity cases as 1 and middle‐aged controls as 0. The variables included diseases, cognition, and lifestyle factors, for example, suffering from a disease or not was defined as 1 and 0, respectively.

### 2.11 Polygenic risk Scores (PRS) analysis

In this cohort study, we calculated weighted polygenic risk scores based on 3,966 known susceptibility markers from the GWAS catalog for many age‐related diseases (Table S5). We imputed the missing risk alleles and corresponding beta weights whenever possible by checking the details in the original reports. Markers were coded additively, and the logarithms of the reported odds ratios were used as weights. All markers were clumped by pairwise linkage disequilibrium (*r* ^2^ > 0.8) prior to constructing the polygenic risk score. Each disease containing at least five SNPs was used to generate the PRS for each individual, and 87 diseases were ultimately included (Table S14). PRS analysis was performed to calculate the correlations of longevity and disease risks not only in all individuals but also in males and females, respectively.

### 2.12 Mendelian randomization (MR) analysis

We had detailed disease records for each individual. Therefore, we performed the GWAS for each of the disease types using the same approach that we applied for the longevity association analysis and used summary statistics data for longevity and various diseases for MR analysis. To calculate the causal effect of longevity on diseases, as well as the effects of diseases on longevity, we performed a bidirectional MR analysis using four different MR methods, including the GSMR method (Zhu et al., 2018) in GCTA tool and inverse‐variance weighting (IVW), weighted median and MR–Egger regression methods implemented in the “TwoSampleMR” R package for robust validation. A consistent effect across the four methods is less likely to be a false positive. In the process of the MR analysis, we selected independent SNPs as instrumental variables, setting a linkage disequilibrium threshold of *r* ^2^ < 0.2 in a 500‐kb window. We explored multiple settings for instrument strength with *p* < 10^−3^, *p* < 10^−4^, and *p* < 10^−5^, respectively. We used the MR–Egger intercept implemented in MR–Egger regression to test for the presence of directional pleiotropy.

### 2.13 Survival analysis

By the last interview, 3,040 of the 12,664 individuals were reported to have died by their families. To study the relationship between genotypes and life span, we used age and live/dead status as phenotypes, and we used a multivariate Cox proportional regression model to perform association analysis. The model was implemented with the “coxph” function from the survival package in R 3.5.1. In the model, the individuals were either dead (status 1), alive (status 0); or missing at the follow‐up interview were subjected to censoring. The surviving subjects were calculated according to age and censoring parameters. Then, the Cox regression was performed using all genotypes and sex as independent variables and the surviving status as the dependent variable. The survival curves were plotted using the “survfit” function in R 3.5.1.

### 2.14 Lasso regression prediction

The prediction analysis was completely independent from the association analyses, and all the 23,769 SNPs incorporated with 19 disease statuses, five lifestyle measurements, and sex were entered into the least absolute shrinkage and selection operator (Lasso). Lasso is a supervised machine learning method that can select a subset of SNPs to achieve the best prediction efficiency. The disease status, lifestyle measurements, and SNP genotypes were imputed separately. Disease status and lifestyles were imputed using the MICE package in R 3.5.1. All the SNPs remaining after QC (*n* = 23,769) were imputed internally without using any reference panel, because the reference panel‐based imputation leverages linkage disequilibrium information (e.g., highly correlated SNPs will be imputed), which is redundant information in terms of prediction. The missing genotypes were imputed using Beagle 5.0 (Browning et al., 2018). Together with sex, 23,824 predictors were entered into the Lasso regression (Tibshirani, 1996). The whole dataset was split into 80% and 20% subsets for model training and testing. Fivefold cross‐validation was conducted for the training dataset. The training process for Lasso regression included feature selection and model fitting. Only one of the features with redundant information was selected for modeling, such as SNPs in high linkage disequilibrium. For longevity prediction, AUCs were calculated to evaluate prediction efficiency. For the life span prediction, the explained variance for life span was estimated using linear model. The predictions were also performed in the male and female groups separately.

## 3 RESULTS

### 3.1 Study subjects and design

**FIGURE 1:**
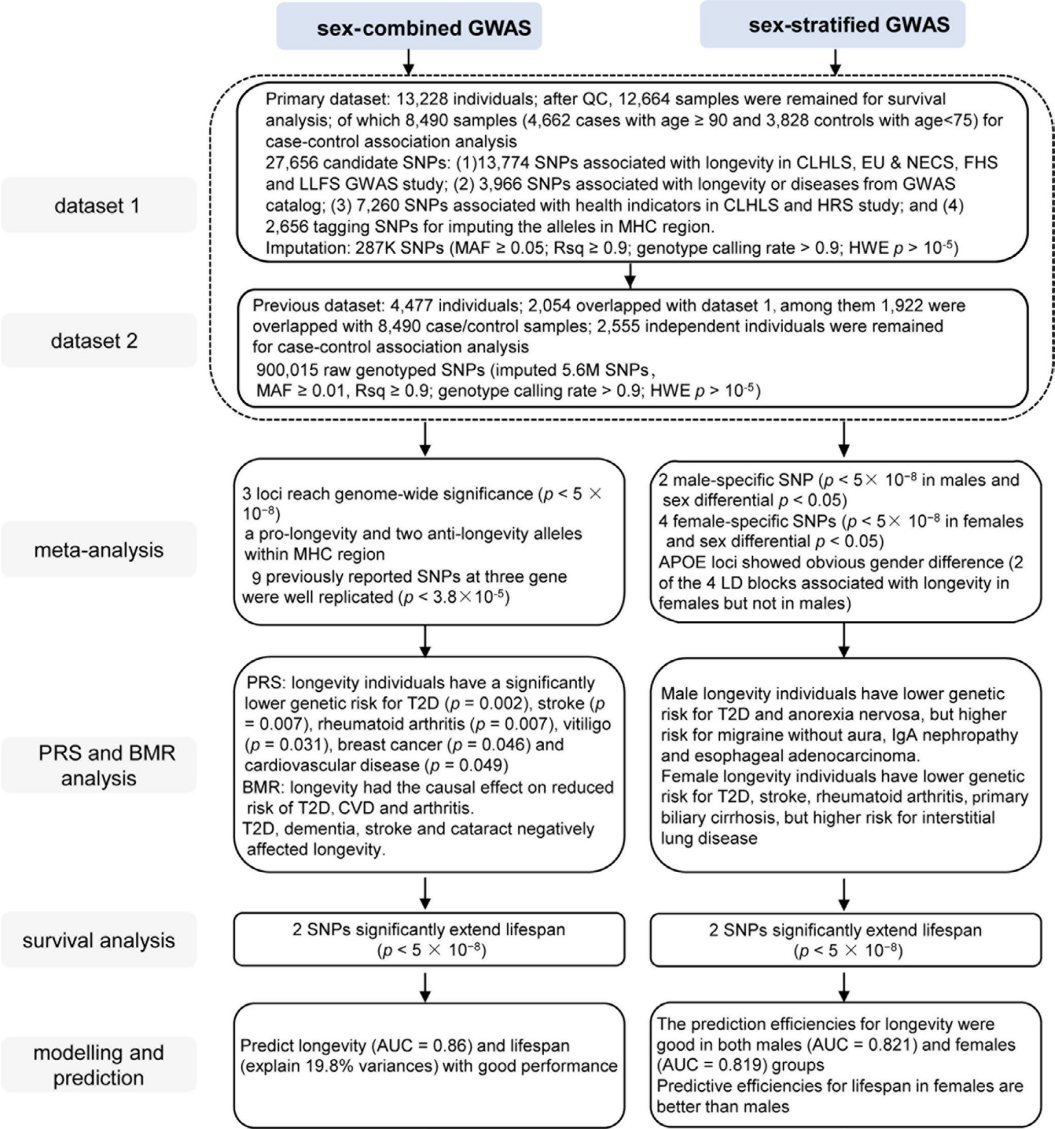
Study design and workflow. To investigate the longevity‐associated genes/pathways, we first carefully selected and designed a customized SNP chip that captured 27,656 candidate variants mainly for longevity as well as disease, health indicator, and immunity. Then, we genotyped these SNPs in a large sample of 13,228 individuals. Next, we carried out the genetic association analyses using age‐stratified phenotype (“cases” were defined as individuals surviving past 90 years of age and “controls” with age less than average life span of 75) as well as incorporating all individuals’ age and the survival status as phenotype, respectively. Furthermore, we performed meta‐analysis together with previous dataset (removing overlapped samples) to identify the longevity genes in gender‐combined and gender‐stratified groups, respectively. In addition, we evaluated polygenic prediction of diseases on longevity using polygenic risk score (PRS) analysis and inferred causal relationships between longevity and diseases using bidirectional Mendelian randomization (MR) method. Finally, we built the prediction model for longevity and life span using all existing factors.

This study was composed of two datasets including a total of 15,651 individuals. The first included 13,228 individuals with 27,656 longevity‐ and disease‐related SNPs genotyped by using our customized SNP chip (Tables [Link], [Link], see also Section 2). After implementing the standard quality control procedures, 12,664 samples and 23,769 out of the 27,656 SNPs remained for subsequent analysis (Figure S1A). Reference‐based imputation enlarged the dataset into 287,000 SNPs. The second dataset was the GWAS set that we previously published, including 4,477 samples (2,178 centenarians and 2,299 middle‐aged controls, Figure S1B) and 5.6 M imputed SNPs (Zeng et al., 2016). The two datasets included 2,054 overlapping individuals, and the genotype concordances of SNPs for the same individual were measured for quality control. The discordant SNPs (genotype concordance <0.9) were removed, and the remaining SNPs were imputed for association analysis. The analysis flow is presented in Figure 1.

We first performed a case–control association analysis on 8,490 individuals (4,662 cases with age ≥90 and 3,828 controls with age <75, Figure S1A) from dataset 1. Twelve SNPs achieved significance after *Bonferroni's* correction (Table S4; *p* < 1.81 × 10^−6^ = 0.05/27,656). In dataset 2, 1,922 individuals, who were overlapped with the 8,490 case/control samples of dataset 1, were removed. We then investigated the significance of these 12 SNPs in the independent dataset 2 with 2,555 individuals. Two SNPs were also nominally significant in the same direction (Table S4; *p* < 0.05). Therefore, we performed both sex‐combined and sex‐stratified meta‐analyses of these two independent datasets including 11,045 individuals to further identify potential longevity genes and pathways. Meanwhile, we carried out survival analysis, polygenic risk score (PRS) prediction, and Mendelian randomization (MR) in all 12,664 individuals from dataset 1. The survival analysis utilized age and the live/dead status as a phenotype to identify SNPs associated with life span. Furthermore, we used PRS analysis and bidirectional MR method to infer causal relationships between longevity and diseases. Finally, we built the prediction model for longevity and life span in 7,807 individuals from dataset 1 with disease status, lifestyle records, and SNP genotypes.

### 3.2 Novel longevity genes revealed by meta‐analysis

**FIGURE 2:**
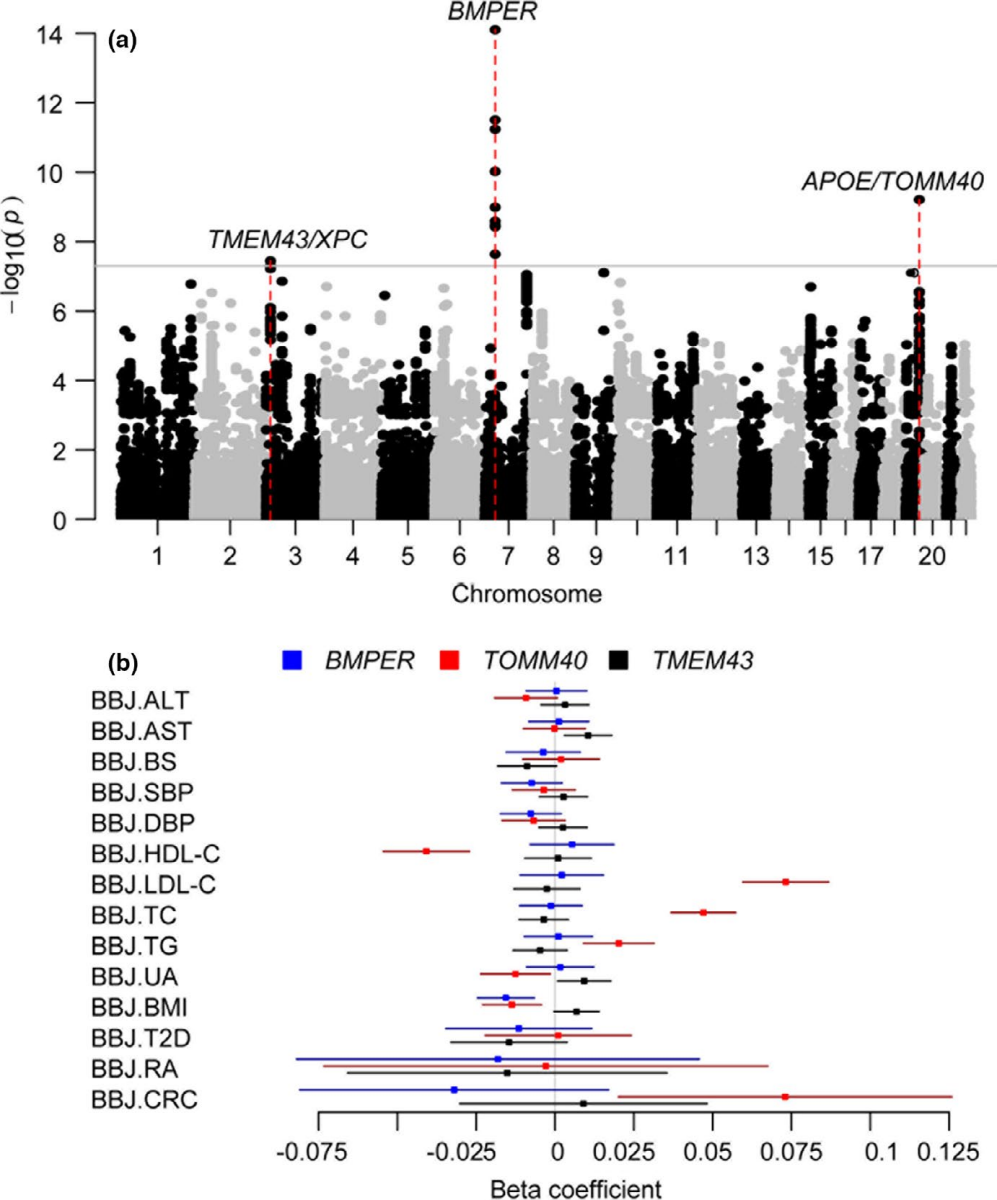
Identification of novel longevity genes by meta‐analysis. (a) Manhattan plot showing the association results by meta‐analysis for longevity. The red line represents a genome‐wide significant *p*‐value (5 × 10^–8^). The gray lines showed the top three loci associated with longevity, including rs17169634 (*p* = 7.91 × 10^−15^) at gene *BMPER*, rs2075650 (*p* = 6.17 × 10^−10^) at *TOMM40/APOE* loci, and rs1043943 (*p* = 3.59 × 10^−8^) at 3'‐UTR of *TMEM43*. (b) Crossing references with GWAS studies from BioBank Japan (BBJ) showed that the three longevity SNPs also linked to multiple traits. ALT, alanine aminotransferase; and CRC, colorectal cancer; AST, aspartate aminotransferase; BMI, body mass index; BS, blood sugar; DBP, diastolic blood pressure; HDL‐C, high‐density lipoprotein cholesterol; LDL‐C, low‐density lipoprotein cholesterol; RA, rheumatoid arthritis; SBP, systolic blood pressure; T2D, type 2 diabetes; TC, total cholesterol; TG, total triglyceride; UA, uric acid.

As shown in Figure 1, we identified three loci that were significantly associated with longevity (*p* < 5×10^−8^) in the meta‐analysis of the two datasets. Among these 3 identified longevity loci, one is the well‐known locus located near the *TOMM40/AOPE* region. The two newly identified genes/loci include *BMPER* and *TMEM43* (Table 1; Figure 2A; Figure S2). The top signal, rs17169634 (*p* = 7.91 × 10^−15^), is located in the intronic region of the *BMPER* gene and has been reported to be associated with Alzheimer's disease (Nelson et al., 2014; Table S5). The second significant signal is the well‐known longevity locus *TOMM40/APOE* (rs2075650; *p* = 6.17 × 10^−10^). The third top SNP, rs1043943 (*p* = 3.59 × 10^−8^), is located in the 3'‐UTR of *TMEM43*, which is in strong linkage disequilibrium (LD) with rs2228001 (*r* ^2^ = 0.95; *p* = 1.13 × 10^−7^), a missense mutation in the *XPC* gene. It has been reported that rs1043943 might regulate the expression of *XPC*, which is a nucleotide excision repair (NER) gene involved in DNA damage repair, and the deletion of *XPC* leads to the development of lung tumors in mice (Hollander et al., 2005). We investigated these three identified longevity‐associated SNPs in the two largest relevant meta‐analyses results (Deelen et al., 2019; Timmers et al., 2019; Table S6). The *TOMM40/APOE* (rs2075650) locus was significant in all three studies and shows consistent effect directions for the minor allele. It is also the most notable genetic loci across many GWAS in multiple ethnical populations. The other two loci were not replicated. Rs1043943 in *TMEM43* was nearly nominal significant (*p* = 0.057) in parents’ life span study by Timmers et al. but not significant in Deelen's study (*p* > 0.5). While for rs17169634 in *BMPER*, although Deelen's study showed nominally significance (*p* = 0.033), the effect of minor allele G was in the opposite direction with our results. We further performed association analyses for diseases in dataset 1 and found that the *BMPER* locus was associated with arthritis (*p* = 3.76 × 10^−6^) and prostate cancer (*p* = 6.32 × 10^−3^), and the G allele for SNP rs17169634 has decreased effects on the risk of arthritis and prostate cancer. *TOMM40*/*APOE* locus linked to dementia (*p* = 2.40 × 10^−4^) and arthritis (*p* = 3.01 × 10^−3^), and *TMEM4*3 was associated with Parkinson's disease (*p* = 0.045). Interestingly, our three longevity SNPs have also been linked to multiple metabolic traits in GWASs on the Japan BioBank dataset (Triendl, 2003; Figure 2B). Specifically, *BMPER* is associated with body mass index (BMI); *TOMM40/APOE* is associated with low‐density lipoprotein cholesterol (LDL‐C), high‐density lipoprotein cholesterol (HDL‐C), total cholesterol (TC), total triglyceride (TG), and colorectal cancer (CRC), and *TMEM43* is associated with aspartate aminotransferase (AST) and blood uric acid (UA).

**TABLE 1:** Three loci associated with longevity at genome‐wide significance

SNP	CHR	BP	Gene	EA	NEA	Dataset 1			Dataset 2 (removing overlapped samples)			Meta‐analysis			Associated diseases
						*β*	SE	*p*	*β*	SE	*p*	*β*	SE	*p*	
rs17169634	7	34093997	*BMPER*	G	A	0.370	0.043	3.87E‐18	−0.097	0.109	0.376	0.308	0.040	7.91E‐15	Arthritis (*p* = 3.76E‐06); prostate cancer (*p* = 6.32E‐03)
rs2075650	19	45395619	*TOMM40*	G	A	−0.261	0.052	6.76E‐07	−0.442	0.111	7.26E‐05	−0.293	0.047	6.17E‐10	Dementia (*p* = 2.40E‐04); arthritis (*p* = 3.01E‐03)
rs1043943	3	14183410	*TMEM43*	T	C	0.165	0.033	4.24E‐07	0.081	0.042	0.016	0.146	0.029	3.59E‐08	Parkinson's disease (*p* = 0.045)

Note

After standard quality control process, 8,490 individuals (4,662 cases with an age ≥90 and 3,828 controls with an age <75; 75 is the average life span of Chinese individuals) in dataset 1 and 2,555 independent individuals (removing 1,922 samples overlapped between 8,490 individuals in dataset 1 and 4,477 individuals in dataset 2; remaining 1,105 centenarians and 1,450 controls with age <65) in dataset 2 were included for case–control association analysis. A meta‐analysis of the two datasets was performed. The BP (base‐pair position) is based on Genome Reference Consortium Human Build 37 (GRCh37).

Abbreviations: EA, effect allele; NEA, not effect allele; SE, standard error.

Since we enriched SNPs located in the MHC region, we next imputed 104 HLA alleles using the HAN‐MHC reference panel (Zhou et al., 2016) and explored their relationships with the longevity phenotype. No SNPs in the HLA region reached genome‐wide significance. While considering HLA alleles, three were observed to be significant after adjusting for the total number of reconstructed HLA alleles (*p* < 0.0005 = 0.05/104, Table S7). Among these three newly identified HLA alleles, HLA‐DQB1*0609 was identified as a pro‐longevity allele (*β* = 0.42; *p* = 3.34 × 10^−4^), while HLA‐C*12:02 and HLA‐B*52:01 were found to be anti‐longevity (*β* < 0). HLA‐C*12:02 is a susceptibility factor in the late‐onset type of psoriasis in Japanese (Mabuchi et al., 2014), while HLA‐B*52:01 has been reported to increase the risks of pulmonary infarction, ischemic heart disease, aortic regurgitation, hypertension, renal artery stenosis, cerebrovascular disease, and visual disturbance (Kitamura et al., 1998).

To further explore the biological functions of these identified longevity‐associated signals, we investigated 300 SNPs associated with longevity at a suggestive significance level (*p* < 10^−5^) using the FUMA tool (Watanabe et al., 2017). Interestingly, we found that these longevity‐associated genes presented significantly up‐regulated expression patterns in multiple specific cerebral regions, including the brain substantia nigra and brain amygdala (Figure S3). Through cross‐referencing with the GWAS catalog, we found that longevity‐associated SNPs showed significant enrichment related to the cerebrospinal total tau (T‐tau) levels, cerebrospinal phosphorylated tau (P‐tau 181p) levels, cerebral amyloid deposition (PET imaging), and various age‐related diseases, such as Alzheimer's disease, age‐related macular degeneration, type 2 diabetes, and ischemic stroke (Figure S4). Based on KEGG analysis, four enriched pathways included type 2 diabetes mellitus, Alzheimer's disease, and the two previously identified calcium and MAPK signaling pathways (Figure S5). Taken together, we found that longevity‐related genes (*TOMM40*, *APOE*, *APOC1*, *PVRL2*, *BRAF*, *MRPS33*, *RYR2*, *BMPER*, *PMF1*, *BGLAP*, *MEIS1*, *CACNA1D*, *ZNRF3*, *CTNND2*, *CELF2*, *KREMEN1*, *ANKRD30A*, *WFS1*, *PPP2R2C*, *GLG1*, *KLHL29*,*WSCD1*, *FUT10*, *TMEM43*) are also implicated in diseases and pathways involved in cardiovascular homeostasis, immunity, inflammation, lipid metabolism, and cognitive function. These associated gene sets are usually clustered with each other, suggesting the anti‐correlation between these age‐related diseases and longevity.

### 3.3 Sex‐specific genes associated with longevity

**FIGURE 3:**
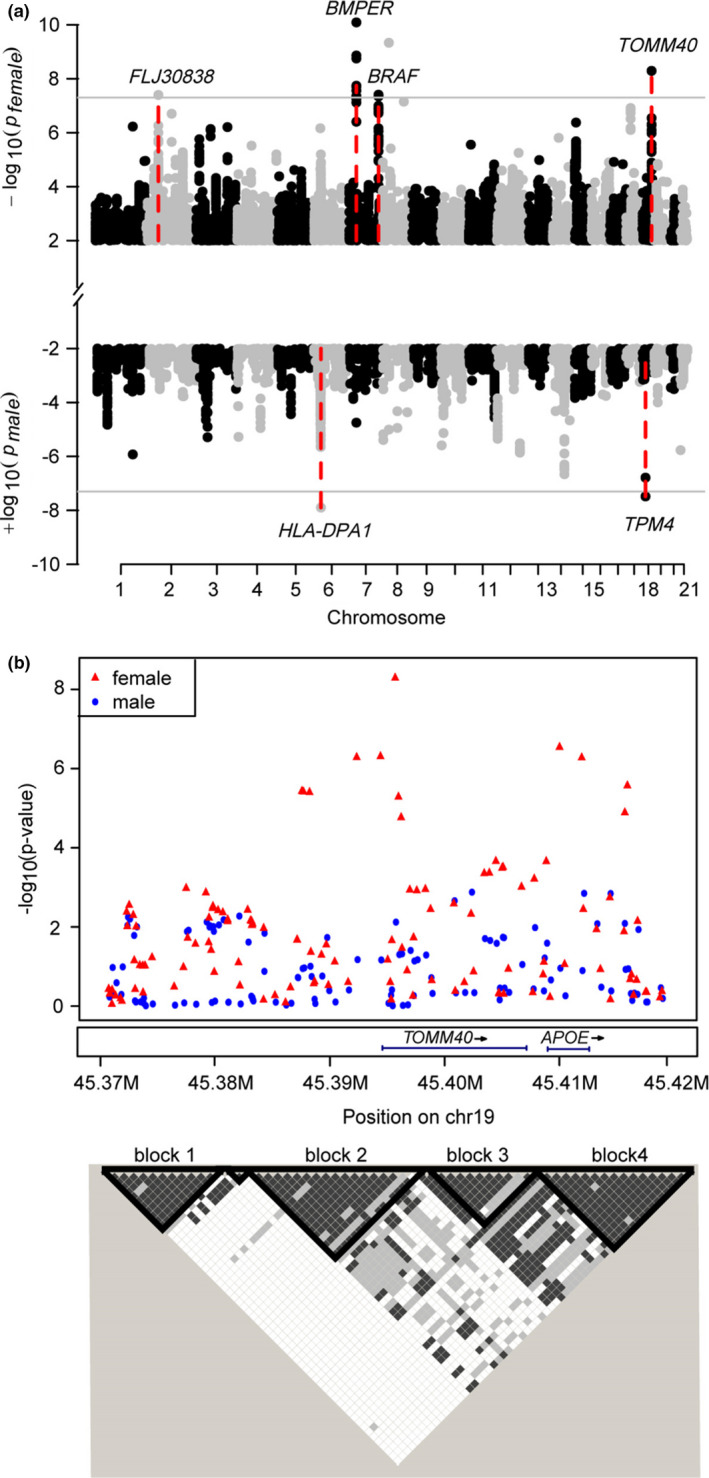
Sex differences in genetic associations with longevity. (a) Manhattan plot showing the male‐specific and female‐specific associations for longevity. The gray line represents the genome‐wide *p* threshold (5 × 10^−8^). Four female‐specific (*FLJ30838*; *BRAF*; *BMPER*; and *TOMM40*) and two male‐specific genes (*HLA‐DPA1* and *TPM4*) were marked in red lines. (b) Regional plot showing the association results for *TOMM40/APOE* locus in males and females, respectively. The LD blocks of this locus were also plotted using Haploview tool, SNPs in block 2 (rs12972156, rs12972970, rs34342646, rs6857, rs71352238, rs2075650, rs34404554) and block 4 (rs769449, rs429358) showed sex differences (*p*_difference_ < 0.05), whereas SNPs in the other two blocks were not.

Regarding the sex difference in genetics of longevity, we performed a sex‐stratified genetic association analysis. We compared the three genome‐wide significant SNPs between sexes and found that only the *APOE/TOMM40* SNP showed a sex difference (Table 2, *p*_male_ = 0.008; *p*_female_ = 5.12 × 10^−9^; *p*_sex difference_ = 0.049). The other two SNPs were not significantly different between males and females (*p* < 0.05 in both males and females, and *p*_difference_ > 0.05). Notably, we identified two male‐specific associations (Table 2; Figure 3A; *p* < 5×10^−8^ in males and *p*_sex difference_ < 0.05) and four female‐specific associations (*p* < 5×10^−8^ in females and *p*_sex difference_ < 0.05) linked to longevity. The male‐specific longevity locus, rs2308910 at *HLA‐DPA1*, was a missense mutation at amino acid position 59 where Glu is converted to Asp. *HLA‐DPA1* as an HLA class II gene plays a central role in the immune system by presenting peptides derived from extracellular proteins. The other male‐specific longevity SNP rs16981095 regulated the expression of the gene *TPM4* in whole blood (Westra et al., 2013) that its related pathways are dilated cardiomyopathy and cardiac muscle contraction. We also found four female‐specific loci for longevity: rs10490092 at *FLJ30838*; rs6967652 at *BRAF*; rs73329134 at *BMPER*; and rs2075650 at *TOMM40/APOE*. Among these female longevity SNPs, rs2075650 had been linked to longevity in multiple studies as well as in our sex‐combined analysis (*p* = 6.17 × 10^−10^). However, we found that rs2075650 is associated with longevity with higher effect in females (*β* = −0.360; *p* = 5.12 × 10^−9^) than males (*β* = −0.197; *p* = 0.008). More specifically, the *TOMM40/APOE* locus (chr19:45.37 M‐45.42 M) could be split into four LD blocks (Figure 3B; Table S8), among which the SNPs in block 2 (rs12972156, rs12972970, rs34342646, rs6857, rs71352238, rs2075650, rs34404554) and block 4 (rs769449, rs429358) showed sex differences (*p*_difference_ < 0.05), whereas the SNPs in the other two blocks did not.

**TABLE 2:** Sex‐stratification results for longevity

SNP	Chr.	position	EA	NEA	Gene	Males						Females						Sex difference
						Dataset 1		Dataset 2		Meta‐analysis		Dataset 1		Dataset 2		Meta‐analysis		
						*β*	*p*	*β*	*p*	*β*	*p*	*β*	*p*	*β*	*p*	*β*	*p*	*p*
Six sex‐specific loci associated with longevity at genome‐wide significant levels																		
Two male‐specific loci																		
rs2308910	6	33037587	a	t	*HLA‐DPA1*	0.878	3.28E‐09	0.107	0.8108	0.802	1.27E‐08	0.141	0.254	0.045	0.8488	0.120	0.2711	1.35E‐04
rs16981095	19	16190863	g	t	*TPM4*	0.455	1.66E‐09	−0.093	0.6584	0.392	3.29E‐08	0.184	8.38E−03	0.063	0.6117	0.155	0.0109	1.09E‐02
Four female‐specific loci																		
rs10490092	2	58892049	c	t	*FLJ30838*	0.123	0.131	0.003	0.988	0.110	0.1537	0.360	2.43E‐06	0.364	6.20E‐03	0.361	4.01E‐08	1.31E‐02
rs6967652	7	140576149	g	a	*BRAF*	0.163	0.0181	−0.366	0.0832	0.112	0.0876	0.451	3.04E‐11	−0.101	0.4049	0.320	3.91E‐08	1.88E‐02
rs2075650	19	45395619	g	a	*TOMM40*	−0.168	0.0305	−0.467	0.04906	−0.197	7.64E‐03	−0.336	1.87E‐06	−0.436	5.64E‐04	−0.360	5.12E‐09	4.87E‐02
rs73329134	7	34071880	t	c	*BMPER*	0.207	7.61E‐03	−0.050	0.812	0.176	0.0155	0.499	9.76E‐12	−0.078	0.5462	0.359	1.82E‐08	3.92E‐02
Sex difference results for the three significant loci identified in sex‐combined association																		
rs17169634	7	34093997	g	a	*BMPER*	0.288	3.36E‐06	−0.122	0.5593	0.255	1.79E‐05	0.444	7.25E‐14	−0.093	0.472	0.350	8.17E‐11	2.34E‐01
rs2075650	19	45395619	g	a	*TOMM40*	−0.168	0.0305	−0.467	0.0491	−0.197	7.64E‐03	−0.336	1.87E‐06	−0.436	5.64E‐04	−0.360	5.12E‐09	4.87E‐02
rs1043943	3	14183410	t	c	*TMEM43*	0.128	8.27E‐03	0.069	0.5636	0.100	0.0254	0.197	9.42E‐06	0.137	0.05323	0.180	1.75E‐06	1.76E‐01

Note

Male‐specific analysis was performed in 4,413 males (3,758 males in dataset 1 and 655 independent males in dataset 2). Female‐specific analysis was performed in 6,632 females (4,732 females in dataset 1 and 1,900 independent females in dataset 2). EA, effect allele; NEA, not effect allele; the sex‐differential *p*‐values tested for difference between the male‐specific and female‐specific beta estimates using the T‐statistic.

We next investigated sex‐specific pathways based on sex‐specific loci. The best‐fit *p* cutoffs of 0.0178 and 0.0004 according to PRSice software were used to select SNPs for pathway analyses in males and females, respectively. The selected longevity‐associated SNPs were significantly enriched in 9 pathways for males (false discovery rate, FDR <0.05; Table S9). These pathways were mainly enriched in DNA replication and mismatch repair‐related pathways including mismatch repair, nucleotide excision repair, base excision repair, and pathways related to amino acid metabolism including ATP‐binding cassette transporters (ABC transporters), beta‐alanine metabolism pathway, arginine, and proline metabolism pathway. In females, 10 pathways were enriched and clustered into the cancer‐related pathway, including glioma pathway, melanoma pathway, chronic myeloid leukemia pathway, JAK‐STAT signaling pathway, B‐cell receptor signaling, and pathways related to the metabolism of terpenoids such as terpenoid backbone biosynthesis, porphyrin and chlorophyll metabolism, valine leucine and isoleucine degradation, and glyoxylate and dicarboxylate metabolism (Table S9).

### 3.4 Replication of previously identified loci for human longevity

For validation purposes, we investigated previously reported 1,881 SNPs in multiple GWAS longevity studies (SNPs resources listed in Table S2). After quality control, 1,305 of the 1,881 SNPs were available for investigation. Nine SNPs in three genes were well replicated in this study and showed statistical significance after multiple testing corrections (Table S10; *p* < 3.8 × 10^−5^ = 0.05/1,305). These three replicated genes include *TOMM40/APOE* (rs2075650, rs6857, rs769449, rs429358, rs405509, rs4420638, and rs7412), *FGD3* (rs4573339), and *AKT1* (rs3803304). Additionally, 106 SNPs were replicated with nominal significance (*p* < 0.05). For example, three previously reported SNPs, rs16972414 (*PIK3C3*, *p* = 0.015), rs6721003 (*SCN7A*, *p* = 0.0037), and rs2149954 (*EBF1*, *p* = 0.047), were replicated (Table S11). However, our previously reported SNPs in gene *IL6* and *ANKRD20A9P* associations (Zeng et al., 2016) did not pass quality control. Therefore, they cannot be replicated.

Notably, we replicated 11 out of the 22 previously reported sex‐specific SNPs but not the rare SNPs (Table S12). These 11 associations were nominally significant in one sex (*p* < 0.05) but not significant in the other sex (*p* > 0.05), and the sex‐differential *p* < 0.05. SNP rs4972778 at *KIAA1715* showed the most significant difference between sexes (*p*_male_ = 0.49; *p*_female_ = 6.62 × 10^−6^; *p*_sex difference_ = 5.13 × 10^−4^).

### 3.5 Observational, PRS, and MR analyses identify the correlations of diseases with longevity

On the basis of detailed questionnaire information including sex, age, diseases, cognition, and lifestyle factors, we systematically analyzed the effects of these factors on longevity. First, the observational correlations of longevity with diseases were investigated (Table S13). Interestingly, we found that the most influential factors related to an increased probability of longevity were being female, exhibiting a lower education level, exhibiting a lower career status, not smoking, not drinking, and an absence of diseases such as hypertension, type 2 diabetes mellitus (T2D), cardiovascular disease (CVD), dyslipidemia, gastroenteric ulcer, arthritis or cholelithiasis. At the organ and tissue aging levels, we found that long‐lived individuals are more likely to suffer from cataracts, glaucoma, and dementia diseases, and to exhibit lower activities of daily living (ADL) and Mini‐Mental State Examination (MMSE) scores. In addition, we found that T2D and CVD were significantly inversely correlated with longevity in females.

Based on a total of 3,966 disease markers from the GWAS catalog genotyped in this study, we constructed the polygenic risk scores (PRS) of each participant for 87 disease traits, and we calculated their correlations with longevity. Seven nominally significant correlations were identified, but none of them passed the multiple testing adjusted threshold (Table S14; *p* < 0.0006). However, we observed that long‐lived individuals tended to present lower polygenic risk scores for T2D (*p* = 0.002), stroke (*p* = 0.007), rheumatoid arthritis (*p* = 0.007), vitiligo (*p* = 0.031), breast cancer (*p* = 0.046), or CVD (*p* = 0.049) with nominal significance (*p* < 0.05).

To investigate whether longevity might have causal effects on these diseases and vice versa, we next applied the bidirectional Mendelian randomization (MR) method. We explored multiple settings for instrument strength (*p* < 10^−3^, *p* < 10^−4^, and *p* < 10^−5^) with strict LD pruning (*r^2^* < 0.2). We first identified the causal effect of longevity on the disease. When using all three different thresholds as cutoff values for instrumental strength, we observed that longevity had a causal effect on reduced risks of CVD (*p*_GCTA‐GSMR_=1.16 × 10^−8^) and T2D (*p*_GCTA‐GSMR_ = 1.91 × 10^−6^). These two causal relationships were shown to be robust when the other three MR tests were performed (*p*_inverse_variance_weighted_ = 2.35 × 10^−9^, *p*_weighted‐median_ =1.75 × 10^−4^, and *p*_MR‐Egger_ = 1.23 × 10^−2^ for decreased CVD risk; *p*_inverse_variance_weighted_ = 1.76 × 10^−6^, *p*_weighted‐median_ = 5.64 × 10^−3^, and *p*_MR‐Egger_ = 3.33 × 10^−2^ for decreased T2D risk), and there was no evidence of horizontal pleiotropy (*p*_egger‐intercept_ > 0.05; Table S15). Moreover, longevity showed direct observational correlations with T2D (*p*_observation_ = 2.54 × 10^−12^) and CVD (*p*_observation_ = 1.58 × 10^−7^; Table 3). Both observational correlations and bidirectional MR confirmed the causal effects of longevity on the lower risks of CVD and T2D. While defining *p* < 10^−3^ as cutoff, longevous individuals showed a causal effect on a decreased risk of arthritis (*p*_GCTA‐GSMR_ = 8.22 × 10^−5^), stroke (*p*_GCTA‐GSMR_ = 2.57 × 10^−3^), and hypertension (*p*_GCTA‐GSMR_ = 3.29 × 10^−3^), presenting no horizontal pleiotropy. Conversely, we also investigated the potential effect of disease on longevity, and we observed that T2D and dementia negatively affected longevity when taking *p* < 10^−3^ as an instrumental cutoff, while stroke and cataract negatively affected longevity when taking *p* < 10^−4^ as the instrumental cutoff (Table S15). Taken together, our series of analyses, including observational correlation, PRS, and bidirectional MR analysis, revealed that long‐lived individuals tend to exhibit a lower genetic risk of T2D, CVD, and arthritis, and in turn that the absence or delayed onset of diseases such as T2D, dementia, and stroke lead ones to live longer and have higher odds to be longevity (Table 3; Table S15).

**TABLE 3:** Observational correlation and bidirectional MR analysis for longevity and diseases

Exposure	Outcome	Observational correlation			Bidirectional MR			MR pleiotropy test (Egger intercept)		
		Estimate	SE	*p*	Estimate	SE	*p*	Egger intercept	SE	*p*
*p* < 10^−3^										
Longevity	Cardiovascular disease	−0.517	0.099	1.58E‐07	−0.1337	0.0234	1.16E‐08	0.0063	0.0113	0.5790
Longevity	Type 2 diabetes	−1.252	0.179	2.54E‐12	−0.1918	0.0403	1.91E‐06	−0.0118	0.0183	0.5196
Longevity	Arthritis	−0.151	0.073	3.95E‐02	−0.0681	0.0173	8.22E‐05	0.0037	0.0098	0.2079
Longevity	Hypertension	−0.317	0.069	4.34E‐06	−0.0485	0.0165	3.29E‐03	0.0045	0.0073	0.5387
Type 2 diabetes	Longevity	−1.252	0.179	2.54E‐12	−0.0307	0.0121	1.11E‐02	−0.0217	0.0345	0.5347
*p* < 10^−4^										
Longevity	Cardiovascular disease	−0.517	0.099	1.58E‐07	−0.1261	0.0366	5.69E‐04	0.0302	0.0208	0.1473
Longevity	Type 2 diabetes	−1.252	0.179	2.54E‐12	−0.1838	0.0628	3.40E‐03	−0.0153	0.0318	0.6310
*p* < 10^−5^										
Longevity	Cardiovascular disease	−0.517	0.099	1.58E‐07	−0.1108	0.0551	1.45E‐02	0.0487	0.0345	0.1649
Longevity	Type 2 diabetes	−1.252	0.179	2.54E‐12	−0.1546	0.0930	1.66E‐02	−0.1059	0.0563	0.0662
Longevity	Arthritis	−0.151	0.073	3.95E‐02	−0.1538	0.0408	1.63E‐04	0.0818	0.0637	0.2057

Note

This table listed significant causal relationships through both observational correlation and bidirectional MR analysis (in the same effect direction and *p* < 0.05 in both two analysis) under different P thresholds. The observational relationship between longevity and diseases was evaluated by multivariable linear regression analysis while adjusted for age and top two principle components. The bidirectional MR in the table was calculated by TCGA‐GSMR method and the directional pleiotropy was evaluated using the MR–Egger intercept implemented in MR–Egger regression. The details are shown in Table S15.

### 3.6 Determinants of life span identified by survival analysis

**FIGURE 4:**
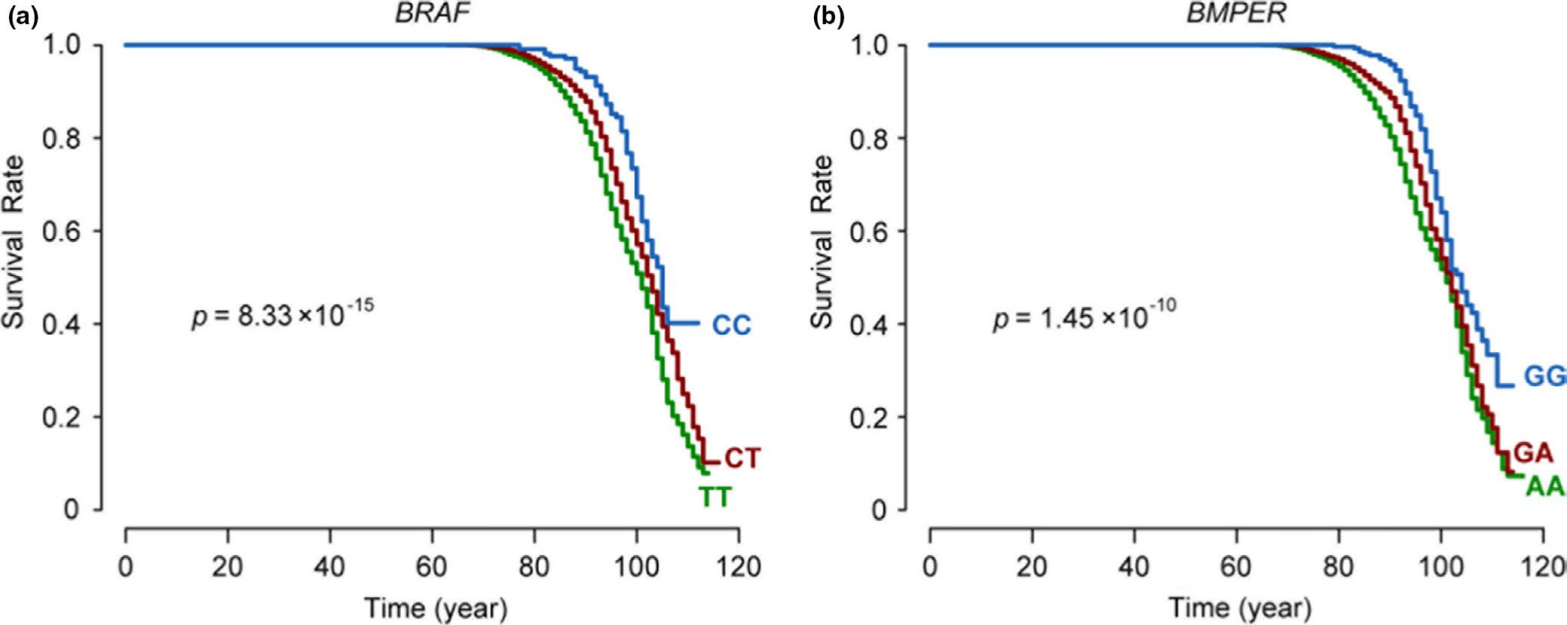
Survival curves for individuals with different genotypes. 12,664 individuals with live/ death status from dataset 1 were included for survival analysis. Approximately 24% (3,040/12,664) of the individuals died with age of death recorded. (a) Survival curves showing the life span of individuals according to *BRAF* gene polymorphism rs1267601. (b) Survival curves showing the life span of individuals according to *BMPER* gene polymorphism rs17169634.

During the follow‐ups, approximately 24% (3,040/12,664) of the individuals died (with an age of death recorded). We performed survival analyses to identify genetic variants associated with life span using the Cox regression model (Table S16). The results showed that rs1267601 in *BRAF* was the variant most associated with life span, and carriers of the CC genotype had significantly higher survival rates at age 100+ compared with CT/TT carriers (28.6% for CC, 16.9% for CT, and 11.8% for TT; hazard ratio (HR) for survival 1.35; *p* = 8.33 × 10^−15^; Figure 4A). In addition, we found that the Alzheimer's disease‐related SNP rs17169634 in *BMPER* was the longevity marker showing the greatest promoting effect, with carriers of the minor allele G exhibiting a substantially longer life span than noncarrier (28.3% for GG, 16.8% for GA, and 11.3% for AA; survival HR = 1.25; *p* = 1.45 × 10^−10^; Figure 4B). Except for the *BRAF* and *BMPER* loci, we did not observe any other loci that reached genome‐wide significance. The *APOE/TOMM40* locus was correlated with life span at nominal significance (*p* = 0.0013).

### 3.7 Predictions of longevity and life span

**FIGURE 5:**
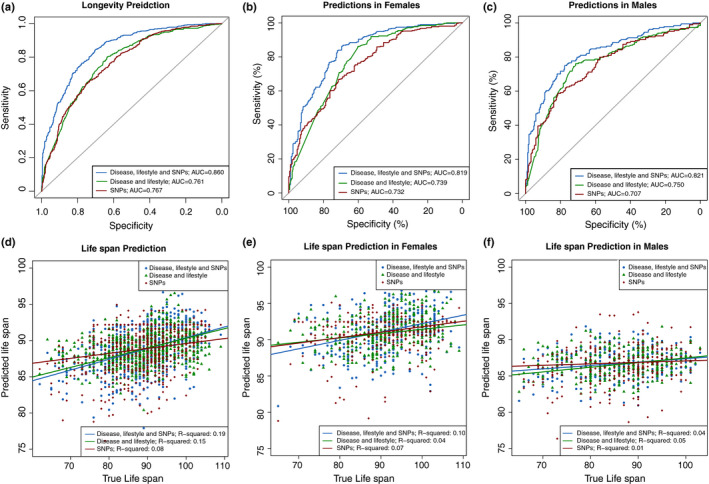
Predictive efficiency for longevity and life span. (a) Receiver operating characteristic curves (ROC) for predictor variables of longevity (binary phenotype). The predictive effectiveness was evaluated by the area under the ROC curve (AUC). The predictive effectiveness of SNPs (AUC = 0.767) was slightly higher compared with disease and lifestyle (AUC = 0.761). The composite SNPs, disease, and lifestyle achieved the best predictive power with AUC = 0.860. ROC for predictor variables of longevity in males (b) and in females (c), respectively. The predictive efficiencies of composite SNPs, diseases, and lifestyles for longevity were presented in both male (AUC = 0.821) and female (AUC = 0.819) groups. The predictive efficiency of SNPs for longevity is 0.732 in females and 0.707 in males. (d) The correlations between true life span and predicted life span showing the predicted effectiveness for life span. SNPs could only explain 2% of the variance for life span, while disease and lifestyle could explain much more variance (15.3%). The composite SNPs, disease, and lifestyle could explain 19.8% of the variance. The predicted effectiveness for life span in males (e) and in females (f), respectively. The predictive effectiveness for life span is better in females than in males. Especially, in males, the SNP set fails to give significant prediction for life span (*p* = 0.10), whereas the selected SNPs could explain 7% of the variance in females (*p* = 1.25 × 10^−6^).

One of the ultimate objectives for identifying factors contributing to longevity is to predict longevity and life span. Based on all the factors we identified from both previous studies and our own association studies, we constructed a predictive model for longevity (age ≥90 vs. age <75) and life span through Lasso regression (Tibshirani, 1996). The prediction was independent of the association study; all the SNPs that we designed on the customized SNP chip (*n* = 23,800 after quality control) as well as 19 disease phenotypes and fivce lifestyles entered prediction model construction. We constructed three models using (1) all SNPs; (2) self‐reported diseases and lifestyles collected in questionnaires; and (3) the combination of all features in models 1 and 2. After removing features and individuals with a high missing rate, the total sample size for the prediction study was 7807. Data were separated into training and testing sets (see Section 2). Models 1 and 2 achieved acceptable discriminations (model 1: AUC = 0.767; model 2: AUC = 0.761). Interestingly, model 1 (using all SNPs) and model 2 (using disease status and lifestyles) yielded similar prediction efficiency. Additionally, the third model, with all the variables, exhibited quite good predictive performances, and the final AUC reached 0.86 (Figure 5A). We further investigated the significance of the SNPs selected by Lasso regression for the prediction in our genetic association study. We found that those SNPs that effectively contributed to the prediction exhibited significantly lower *p*‐value enrichment (Figure S6).

For the prediction of life span, we used 3,023 individuals who had an exact age of death and detailed phenotypic records in our datasets. All three predictive models yielded good performance (Figure 5; model 1: *p* = 1.9 × 10^−5^; models 2 and 3: *p* < 2.2 × 10^−16^). Model 1 could only explain 8% of the variances in the life span, while model 2 could explain 15.3%. Furthermore, model 3, including all features, could explain 19.8% of the variances.

The genetic architectures of longevity and disease progression are quite different between sexes. Next, we applied our predictive models to the male and female groups separately. The predictive efficiency for longevity (binary phenotype) was good in both the male (AUC = 0.821) and female (AUC = 0.819) groups (Figure 5b,c). However, the predictive efficiency for life span in females was superior to that of males (Figure 5e,f). In particular, in males, the SNP set failed to give a significant prediction for life span (*p* = 0.10), while in females, the selected SNPs could explain 7% of the variance (*p* = 1.25 × 10^−6^), indicating that the sex groups indeed exhibited different genetic architectures for aging. The selected features and their coefficients for all prediction models are listed in Tables S17 and S18.

## 4 DISCUSSION

In this study, we present several findings regarding the genetic contributions to longevity and their gender differences based on 15,651 individuals from the cohort of the Chinese Longitudinal Healthy Longevity Survey. We designed an informative SNP chip for studying the genetics of longevity. Longevity case–control analysis (*n* = 11,045) and survival analysis (*n* = 12,664) were performed in different subsets of the cohort. In addition to previously published longevity‐related studies, we included SNPs for relevant diseases, such as CVD and T2D, intending to obtain results that were comparable to those of previous studies. The main findings and several highlights of this work are described below.

First, we identified two novel loci (*BMPER and TMEM43/XPC*) and replicated three loci (*TOMM40/APOE*, *FGD3*, and *AKT1)* associated with longevity in Chinese populations. Interestingly, these five longevity‐associated loci have been linked to diseases, especially age‐related diseases. For example, *BMPER* has been associated with aging and its related diseases, such as Alzheimer's disease (Nelson et al., 2014), and it is also involved in the regulation of the proinflammatory phenotype of the endothelium (Helbing et al., 2011), functioning primarily in the vascular (Lockyer et al., 2017) and respiratory systems (Helbing et al., 2013). *XPC* is involved in DNA damage repair and is associated with disease characterized by an extreme sensitivity to ultraviolet rays from sunlight, such as xeroderma pigmentosum, complementation group c and xeroderma pigmentosum, variant type, and the deletion of *XPC* leads to lung tumors in mice (Hollander et al., 2005). The *TOMM40/APOE* locus has been reported to be associated with longevity in multiple studies among diverse populations and the locus contributes to Alzheimer's disease (Seshadri et al., 2010), age‐related macular degeneration (Cipriani et al., 2012), cardiovascular disease (Middelberg et al., 2011), cognitive decline (Davies et al., 2014), immunity (Reiner et al., 2008), and lipid metabolism/dyslipidemia (Aulchenko et al., 2009). *FGD3*, a putative regulator of cell morphology and motility, was associated with longevity in the NECS study, and its expression plays a prognostic role in breast cancer (Renda et al., 2019). *AKT1* is relevant to longevity (Deelen et al., 2013; Nojima et al., 2013), and the dysregulation of AKT signaling leads to diseases for which there are major unmet medical needs, such as cancer, diabetes, and cardiovascular and neurological diseases (Hers et al., 2011). The two novel signals were also linked to multiple age‐related phenotypes not only in this cohort but also in GWASs from the Japan BioBank, whose cohort is ethnically closer to the Chinese population. The *BMPER* locus was associated with arthritis, prostate cancer, and BMI. The *TMEM43* locus was associated with Parkinson's disease, AST, and UA. These findings consistently revealed the genetic overlap between exceptional longevity and age‐related diseases and traits (Fortney et al., 2015). It is noted that five SNPs including rs10757274, rs4977574, rs2891168, rs10965235, and rs944797 located in well‐known *CDKN2B* locus, which is associated with CVD, were also associated with longevity with nominal significance in our study.

While comparing our current results with other GWAS meta‐analyses and our previous results, inconsistencies were found. SNP rs17169634 in *BMPER* showed different directions of effects in multiple studies. One possible reason could be that the true causal variants are hidden in this area, but due to different linkage disequilibrium (LD) structures among European and Chinese populations, the alleles tagging true causal variants may be different. Future fine‐mapping with denser makers or genome sequencing will be required to illuminate the hidden information. SNP rs17169634 was not significantly associated with longevity in our dataset 2; therefore, the direction of effects in dataset 2 could not be determined that the confidence interval of effect size included zero. The significant signal was driven by dataset 1, where we also tested its association with complex diseases. The G allele for rs17169634 in *BMPER* has reduced effects on the risk of arthritis (*p* = 3.76 × 10^−6^) and prostate cancer (*p* = 6.32 × 10^−3^). Taken together, the results in our data showed that the G allele for rs17169634 in *BMPER* increased the probability of being longevity in our logistic regression and has increased effects for life expectancy in survival analysis and reduced the risks of age‐related diseases. Notably, the causal effects from an increased chance of longevity to reduced risk of arthritis were also identified in our MR analysis. These directions of effects are as expected that long‐lived individuals show a delay in overall morbidity through having beneficial effects for diseases (Andersen et al., 2012). As for our previously reported SNPs in *IL6* and *ANKRD20A9P*, they did not pass the quality control in our current analysis. Therefore, they cannot be replicated. We further checked the frequency of reported SNPs on these two genes. The minor allele of rs2069837 in *IL6 *has a lower frequency (0.075) than it is in the dbSNP Asian population (0.179), and this allele has reduced effects for longevity. Since the proportion of centenarians is much higher in our previous study (48%; Zeng et al., 2016) than in other GWAS studies for longevity, the underrepresentation of this allele in our dataset is plausible. The inconsistency of the results could be caused by the differences in proportions of centenarians between our two datasets and also among different ethnic populations. As for SNP rs2440012 in *ANKRD20A9P*, the minor allele G was overrepresented in our previous study (0.076) compared with Asians in dbSNP (G = 0). It has been filtered out in Deelen's meta‐analysis may be due to multi‐allelic problems in the European population (C = 0.90, A = 0.0015, G = 0.098). Additional independent datasets are needed for a detailed look into these loci.

One existing major problem of longevity genetic studies is that the findings from different studies are difficult to replicate. The reasons could be ethnic differences in genetic background and variation of phenotype definitions. Furthermore, when considering the complex relationships between age‐related diseases and longevity (Andersen et al., 2012; Ukraintseva et al., 2016), the health management systems and culture could also introduce distinctions among populations. For instance, if the age‐related diseases could be managed well, the patients could still survive longer. Therefore, the longevous individuals are mixed with people who carry true protective alleles for the disease and individuals who accepted excellent health care. While the healthcare systems are quite different among countries, especially for older generations, the frequency of longevity‐related alleles could be different in the elderly. Hence, further studies should also take health care and lifestyle into account when classifying cases and controls for comparisons.

Second, we found some sex‐specific loci related to longevity. Numerous studies have reported remarkable sex differences in longevity and life span (Austad & Fischer, 2016; Candore et al., 2006; Ostan et al., 2016; Yuan et al., 2020); however, very few studies have reported the sex‐differential effect of genetics for longevity. *TOMM40/APOE* is well‐characterized longevity locus that could be split into 4 LD blocks. We found that two of these 4 LD blocks were associated with longevity in females but not in males (*p*_difference_ < 0.05), in line with our previous study (Zeng et al., 2018). This may indicate sex‐specific genetic associations of longevity may be caused by differences during meiosis between males and females. The distinction of recombination rates between sex groups has been reported in both human and animals (Li & Merila, 2010; Tapper et al., 2005). Since the recombination was closely interacted with natural selection (Schumer et al., 2018), differences in recombination are plausible to lead to sex or population stratification and thereby causing a small group of people having enriched evolutionary benefit alleles. Therefore, it is necessary to use strand‐specific, long‐segment sequencing technologies or family studies to detailed look into the LD structure for longevity people in future studies.

Interestingly, the predictive effectiveness of SNPs for longevity is slightly better in females (AUC = 0.732) than in males (AUC = 0.707). For life span predictions, SNPs could explain 7% of the variance for life span in females (*p* = 1.25 × 10^−6^) but failed to provide a significant prediction for life span in males (*p* = 0.10). All these results are consistent with our previous finding (Zeng et al., 2018) that the genetic association with longevity is stronger in females than in males. Notably, we found that some diseases also presented sex‐differential patterns associated with longevity. For example, T2D and CVD were more significantly correlated with longevity in females. Previous studies have reported sex differences between cardiovascular diseases and aging, in which it is assumed that genetic traits and sex hormones play the key roles (Rodgers et al., 2019).

Our PRS and MR analyses revealed negative correlations between longevity and multiple diseases, including CVD, T2D, and arthritis. The results were generally in consistent with those in a meta‐analysis of the European population (Joshi et al., 2017). However, other studies indicated different conclusions. One publication based on the Leiden Longevity Study (LLS) suggested that disease risk alleles do not compromise human longevity (Beekman et al., 2010). The authors only considered 30 disease risk SNPs, while our analyses included more carefully selected SNPs for age‐related diseases (Erikson et al., 2016), and the obtained polygenic risk scores reflected an overall significant decrease in genetic disease risk in exceptionally long‐lived individuals. Taken together, these findings suggested that some disease risk SNP alleles might increase the chance of longevity (McDaid et al., 2017), but there are more effective disease risk SNP alleles associated with earlier mortality (Erikson et al., 2016; Joshi et al., 2017). The benefits of utilizing polygenic risk scores are that it summed the effects of multiple alleles instead of looking at the count of each risk allele. Therefore, when considering the additive effects aggregating all risk alleles, the genetic risks of multiple diseases were found to be reduced in long‐lived populations.

There is growing interest in predicting the risks for diseases and complex traits using polygenic risk scores (Khera et al., 2018). Previous studies have predicted longevity and life span based mainly on animal models (Huang et al., 2004; Shen et al., 2014; Swindell et al., 2008) or the use of single biomarker (Ho et al., 2019; Whittemore et al., 2019). One recent study using the UK Biobank dataset applied polygenic risk score for life span prediction with good performance but was limited by utilizing summary statistics for life span based on parental data (Timmers et al., 2019). Our newly developed prediction models including genetic markers performed nicely in the classification of longevity (AUC = 0.767) but were not very effective in the prediction of life span (explaining only 15.3% of the variance). When disease status and lifestyle information were added, the longevity‐ and life span‐prediction models produced better predictions for both longevity (AUC = 0.86) and life span (explaining 19.8% of the variances). Additionally, for the classification of longevity, the performance of SNPs is similar to that of diseases and lifestyles, indicating that genetics and phenotypes may have independent components that influence aging.

A limitation of the present study is the candidate‐gene approach, which might preclude the discovery of new possible causative genes or biological pathways. However, our selection of candidates was primarily based on our previous genome‐wide association studies conducted in 4477 Chinese individuals from the Chinese Longitudinal Healthy Longevity Survey (CLHLS). The SNPs with *p*‐values smaller than 0.015 in our previous GWAS were all selected for inclusions on our customized chip. We collected additional candidate SNPs from existing studies, including studies not only on longevity genetics but also on other age‐related complex diseases and traits. Moreover, by leveraging on imputation technology, the candidate SNP sets were further expanded. By incorporating all these SNPs together, we performed multi‐candidate genes association analyses, which is suboptimal for genome‐wide associations but still very informative. Secondly, in order to identify sex‐specific genetic markers associated with longevity, we stratified our sample into male and female groups. The benefit of stratifying the sample is an increased chance to find those sex‐specific SNPs tagging different causal variants in different sex groups. However, this strategy also has the drawback that the reduced sample size for each analysis group caused decreased power. We only replicated half of the previous identified sex‐specific loci, and more replication studies are required in the future. Thirdly, we noted that the predicted life span was generally shorter than the true life span, indicating undefined missing confounders contributing to the life span (genetic and other confounding factors or their interactions). Future genetic studies of longevity based on affordable exome and whole‐genome sequencing might be helpful to further identify a larger number of longevity‐associated genetic variants by applying the analysis of rare genetic and copy number variants. Together, these findings provide a benchmark for the development of longevity‐ and life span‐predictive models. Further studies are warranted to improve the models through the identification of an additional panel of predictive variables and the development of innovative computational approaches.

In summary, our results not only identified novel longevity genes but also depicted the landscape of genetic contributors to longevity and life span through a complex of sex‐differential and disease‐related interactive circuits, which could be more precisely predicted in the near future.

## CONFLICT OF INTEREST

We declare no competing interests.

## AUTHOR CONTRIBUTIONS

YZ, CN, JM, and XX designed and supervised the study. Xiaomin Liu, ZS, and YL analyzed the data. Xiaomin Liu, ZS, YL, YY, and JM interpreted the data. MF, CB, PA, HC, ZC, BT, JS, XG, MZ, PC, TZ, HJ, Xiao Liu, YH, HY, JW, and FW were involved in data acquisition, collection of phenotype data, genotyping of the individuals, and data management. Xiaomin Liu, ZS, YL, YY, and JM wrote the manuscript. All authors read and approved the final version of the manuscript.

## Supporting information

Fig S1

Table S1

Table S2

Table S3

Table S4

Table S5

Table S6

Table S7

Table S8

Table S9

Table S10

Table S11

Table S12

Table S13

Table S14

Table S15

Table S16

Table S17

Table S18

## Data Availability

The data that support the findings of this study have been deposited and controlled in the CNSA (https://db.cngb.org/cnsa/) of CNGBdb with accession code CNP0000792. The data are currently not publicly available.
