# Supplementary material for: Integrated genetic analyses revealed novel human longevity loci and reduced risks of multiple diseases in a cohort study of 15,651 Chinese individuals
Source: Aging Cell. 2021 Mar 3;20(3):e13323. doi: 10.1111/acel.13323 (PMC7963337; doi:10.1111/acel.13323)
Supplement: Supplementary file 1 — Fig S1 [file ACEL-20-e13323-s010.docx]

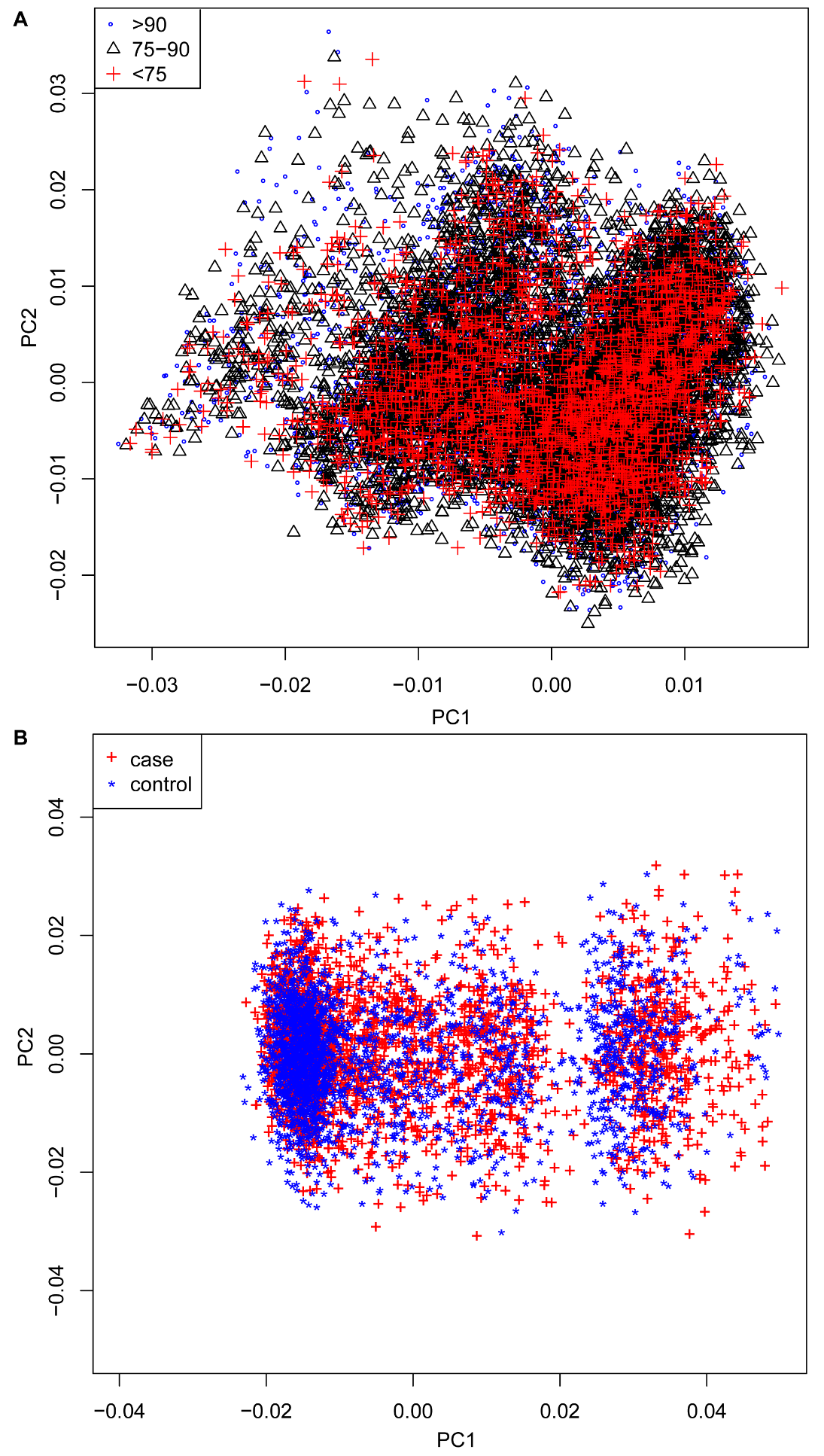


**Figure S1**. **Results of** **multi-dimentional scaling analysis (MDS) in the cases and controls samples for dataset 1 and dataset 2.** MDS was performed on the **(A)** 12,664 individuals (4,662 cases with age >90, 4,174 elder individuals with age 75-90 and 3,828 controls with age<75) in the primary dataset (dataset 1); and **(B)** 4,477 individuals (2,178 cases and 2,299 controls) in the previous dataset (dataset 2).

**

**

**Figure S2**. **Regional plot of the top three signals, including *BMPER*, *TOMM40/APOE* and *TMEM43/XPC*.** For all plots, they are plotted using online LocusZoom tool (http://locuszoom.org/genform.php?type=yourdata). Each point represents a SNP, where the x axis represents the position of the SNP and the y axis represents the –log_10_ *p*-value of the genome-wide association results. Each point is color-coded with the D’ value as calculated in ASN population from the 1000genome Phase3 project. The recombination rate is also plotted at each genomic position with the rate indicated on the y axis (right).

**
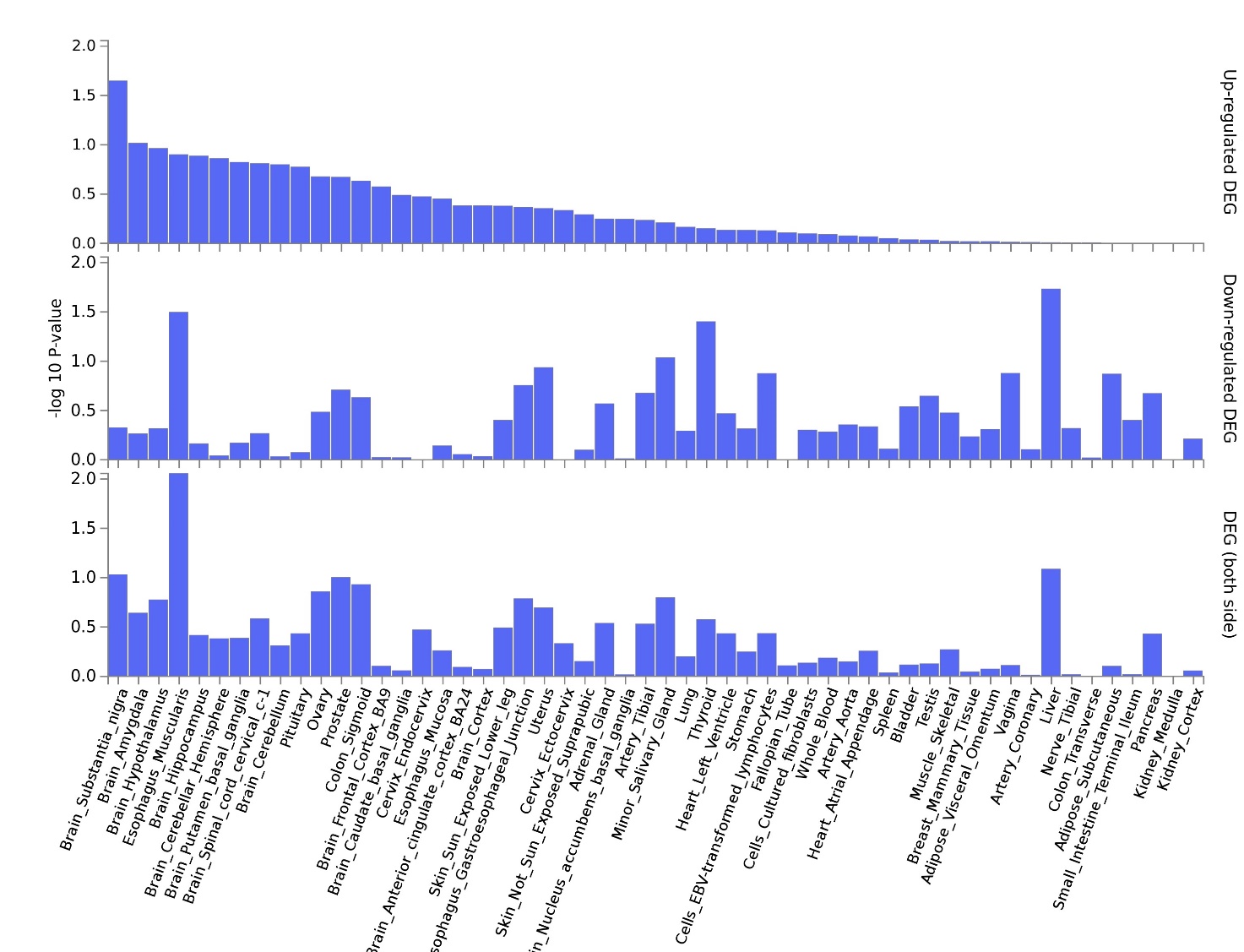
**

**Figure S3. Significantly enriched differentially expressed gene (DEG) sets (*p_adjusted_* < 0.05) of longevity SNPs.** The significant loci with *p* < 10^-5^ identified in gender-combined longevity genetic association analysis were mapped to genes using SNP2GENE in FUMA (http://fuma.ctglab.nl/). We first used the positional mapping method and maps variants to genes based on physical distance within a 20kb window. Mapped genes were further investigated using the GENE2FUNC procedure, which provides hypergeometric tests of enrichment of the list of mapped genes in 53 GTEx tissue-specific gene expression sets.

**
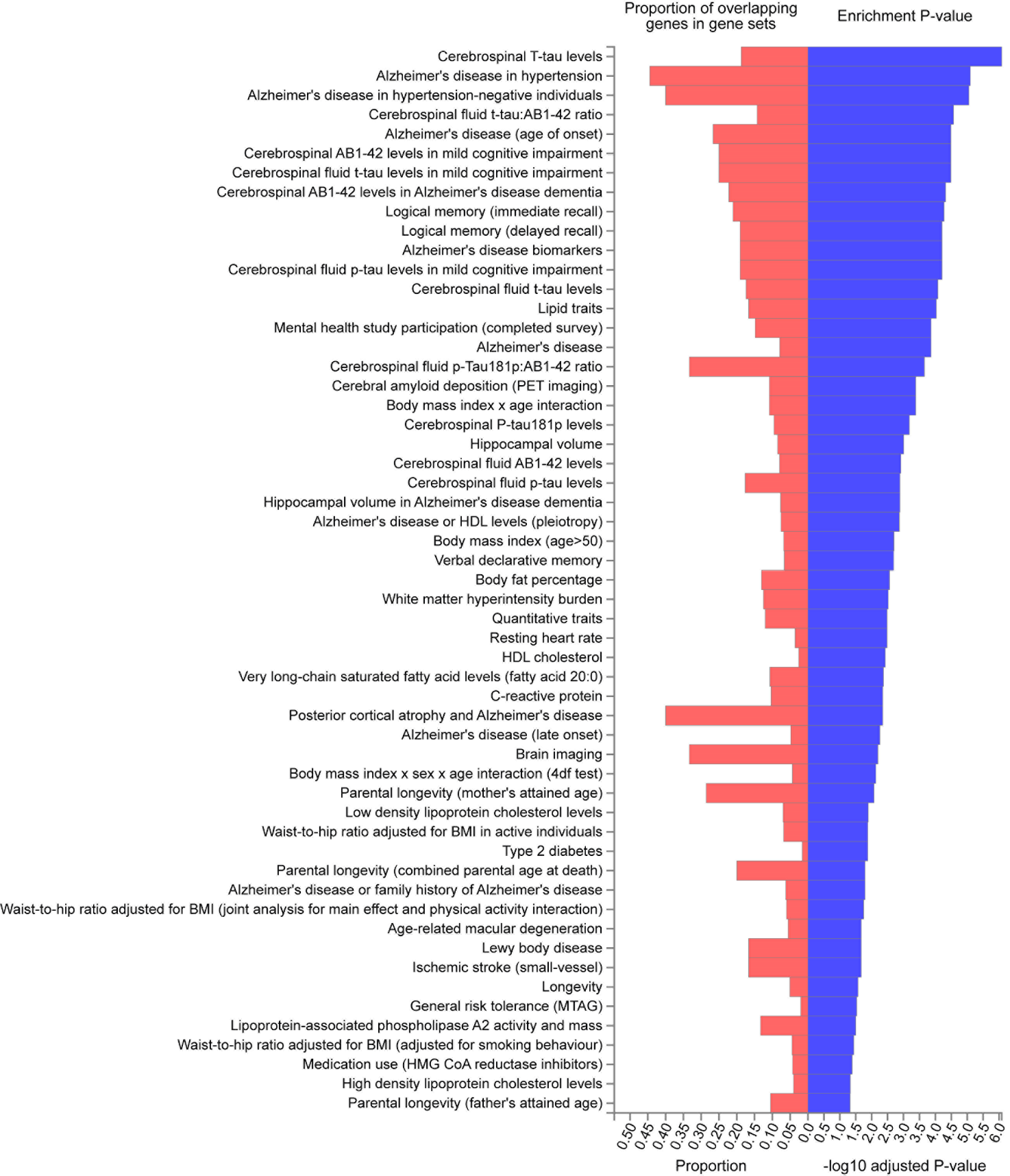
**

**Figure S4. Significantly enriched traits or diseases identified using longevity genes mapped in GWAS catalog (*p_adjusted_* < 0.05)**. The significant loci with *p* < 10^-5^ identified in gender-combined longevity genetic association analysis were mapped to genes using SNP2GENE in FUMA (http://fuma.ctglab.nl/). We first used the positional mapping method and maps variants to genes based on physical distance within a 20kb window. Mapped genes were further investigated using the GENE2FUNC procedure, which test if genes of interest are overrepresented in GWAS catalog.


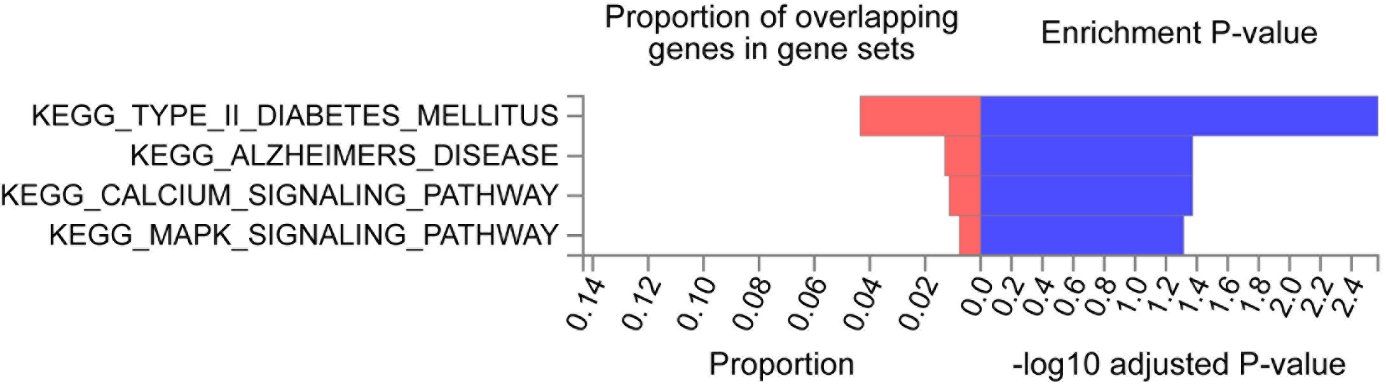


**Figure S5. Enriched KEGG pathways identified for longevity genes (*p_adjusted_* < 0.05).** The significant loci with *p* < 10^-5^ identified in gender-combined longevity genetic association analysis were mapped to genes using SNP2GENE in FUMA (http://fuma.ctglab.nl/). We first used the positional mapping method and maps variants to genes based on physical distance within a 20kb window. Mapped genes were further investigated using the GENE2FUNC procedure, which test if genes of interest are overrepresented in KEGG (MsigDB c2) dataset.


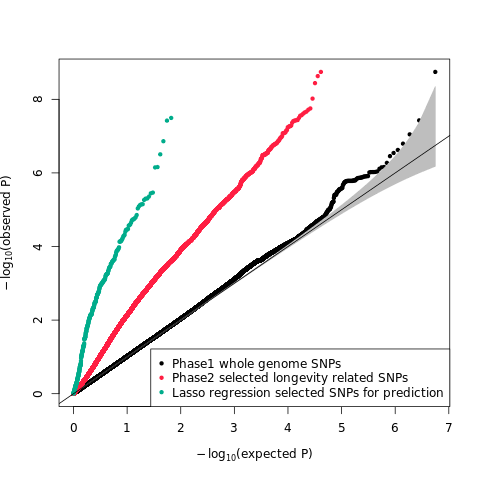


**Figure S6. The QQ plots for the discovery SNPs, replication SNPs and the SNPs selected by lasso regression for prediction of longevity in this study.** Those SNPs effectively contributed to the prediction have significant small P-value enrichment in our GWAS result.


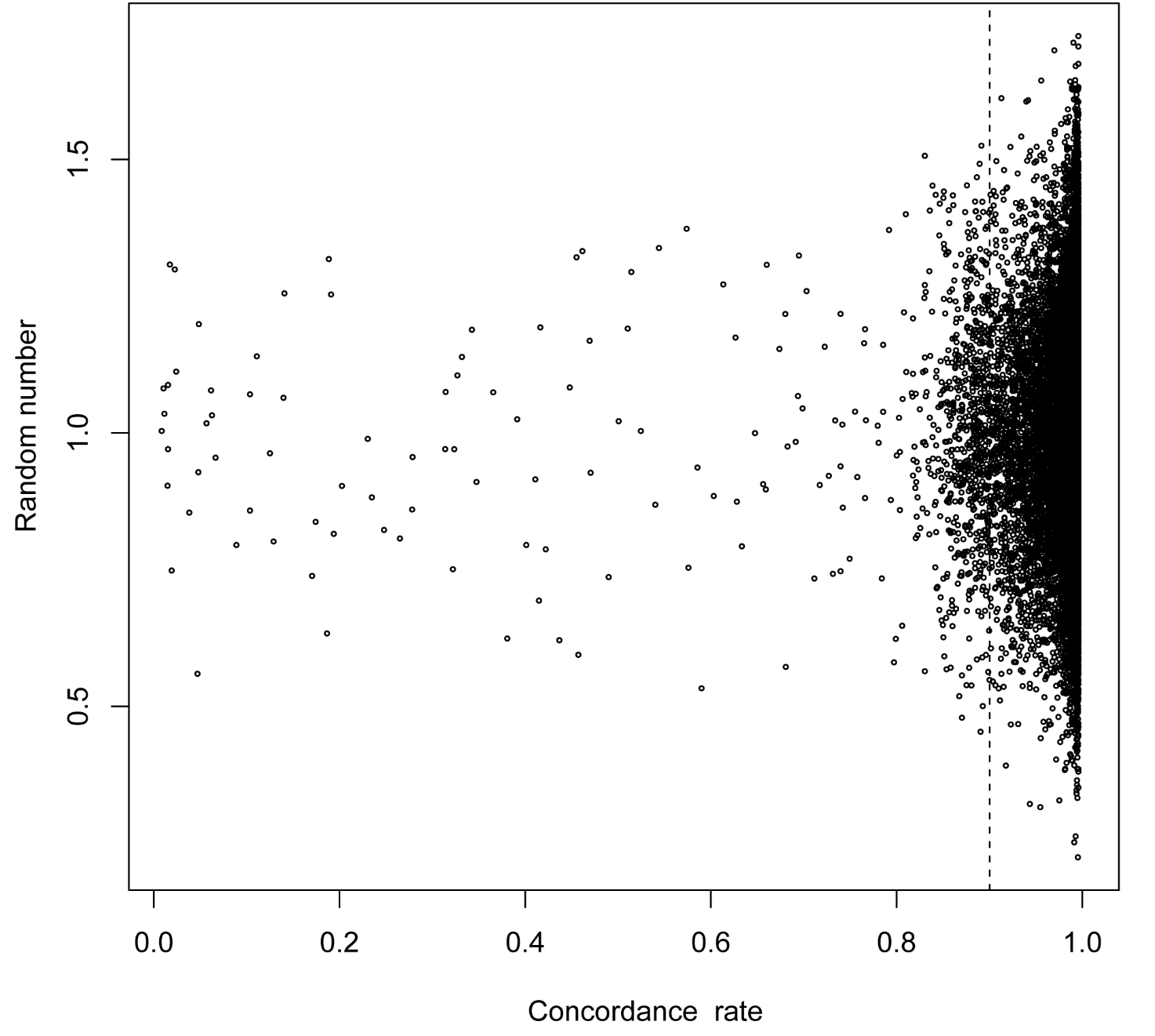


**Figure S7.** **The concordance rate of genotypes in overlapped 2,054 samples genotyped using two different arrays (Affymetrix chip in discovery and Illumina Zhonghua chip in replication).** The genotypes were removed if the concordance rate was lower than 0.9 (dotted black line).
